# Supplementary material for: Interpretable single-cell factor decomposition using sciRED
Source: Nat Commun. 2025 Feb 22;16:1878. doi: 10.1038/s41467-025-57157-2 (PMC11846867; doi:10.1038/s41467-025-57157-2)
Supplement: Supplementary file 1 — Supplementary Information [file 41467_2025_57157_MOESM1_ESM.pdf]

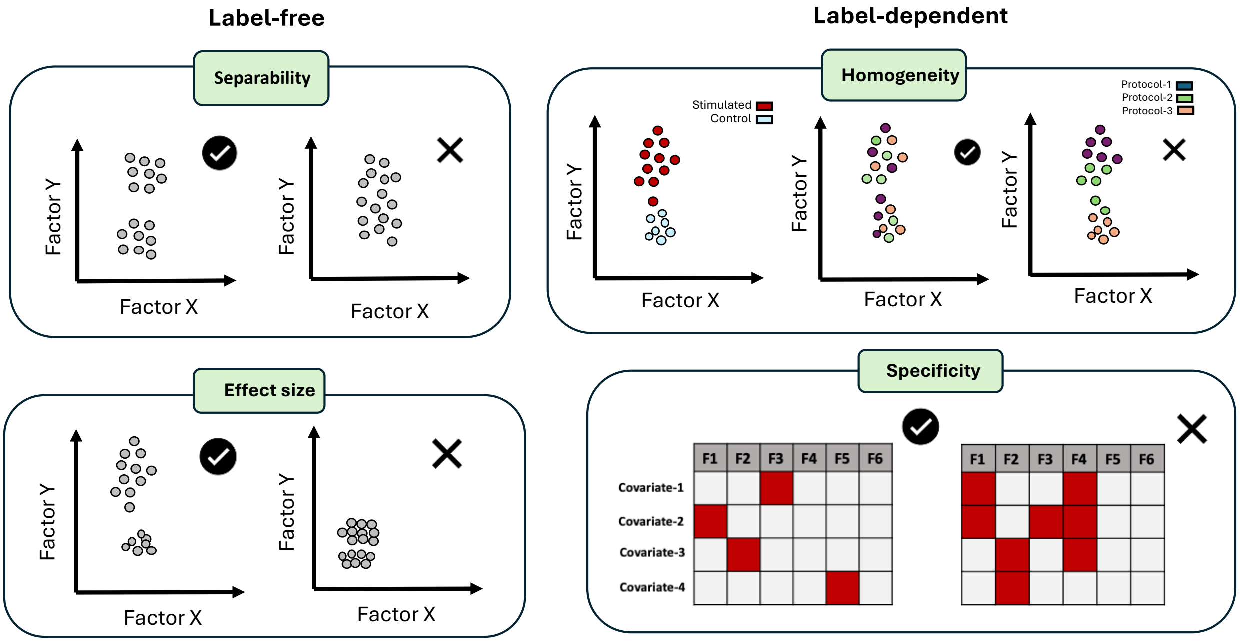

Supplementary Figure 1) Schematic overview of factor interpretability metrics. The four categories of factor interpretability metrics: separability, effect size, specificity, and homogeneity. Separability and effect size are label-free metrics, while specificity and homogeneity are label-dependent.

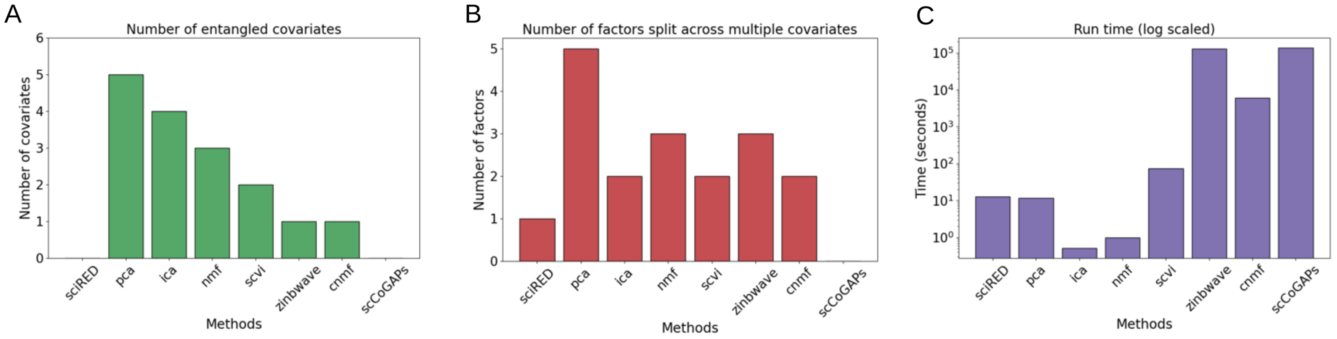

Supplementary Figure 2) Benchmarking sciRED's factor discovery on the scMixology dataset. We evaluated sciRED and seven other factor analysis methods (PCA, ICA, NMF, scVI, Zinbwave, cNMF, scCoGAPs) using the scMixology benchmark dataset to test factor discovery performance in single-cell data with well-defined biological and technical signals. Four evaluation metrics were used to assess performance across methods: A) the number of entangled covariates, indicating covariates matched to multiple factors; B) the number of factors distributed across multiple covariates; C) runtime; and the number of covariate levels without an associated factor. Lower values for all metrics indicate better performance. sciRED outperformed other methods, with the exception of scCoGAPs, on minimizing entangled covariates and factors split across multiple covariates. The log-scaled runtime analysis highlights sciRED's high scalability. scCoGAPs and Zinbwave showed particularly slow run time performance, requiring over 38 and 35 hours, respectively, to process the dataset. All methods successfully captured each covariate level, performing equally well on this metric.

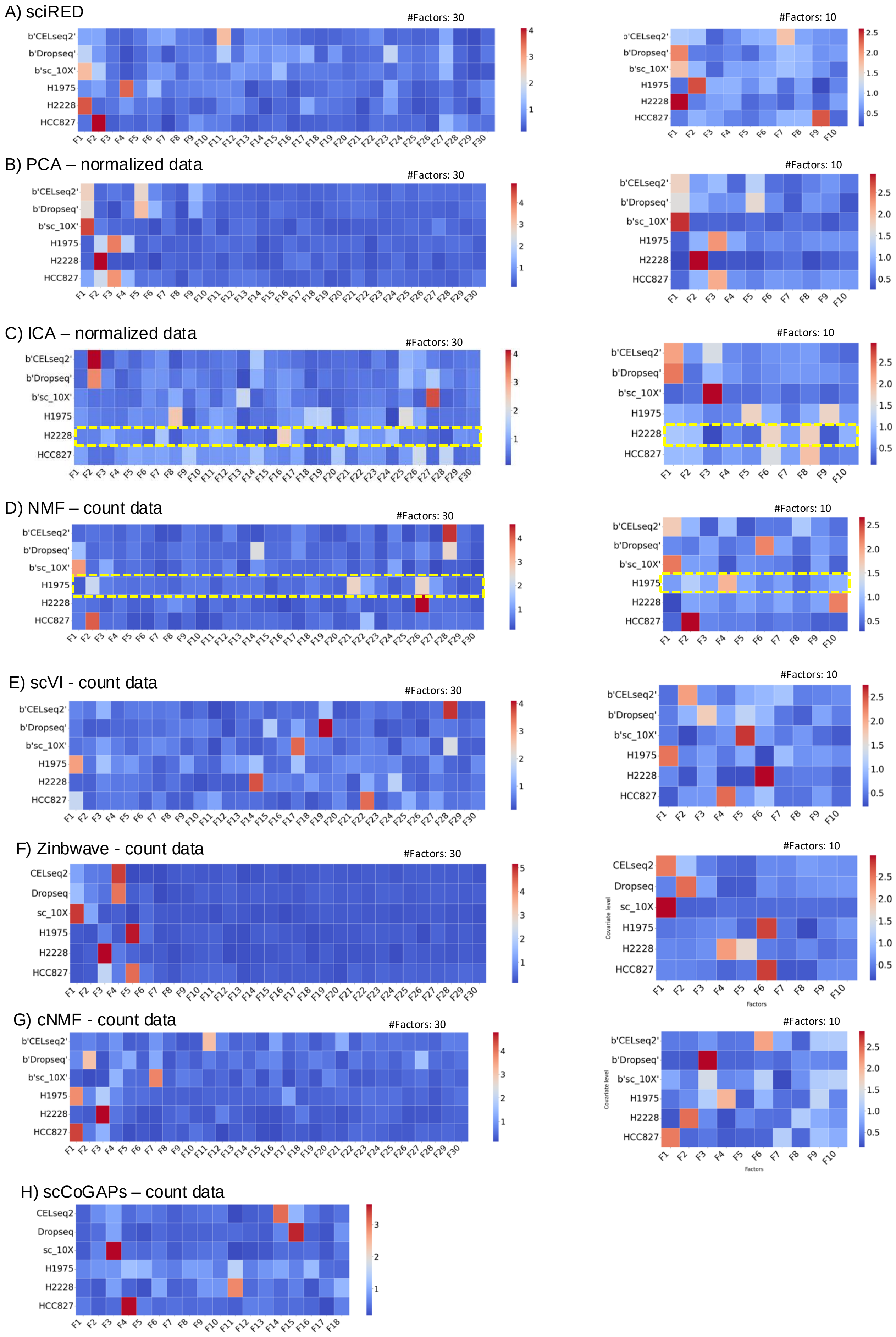

A) sciRED
#Factors: 30
#Factors: 10
b'CELseq2'
b'Dropseq'
b'sc_10X'
H1975
H2228
HCC827
B) PCA – normalized data
#Factors: 30
#Factors: 10
C) ICA – normalized data
#Factors: 30
#Factors: 10
D) NMF – count data
#Factors: 30
#Factors: 10
E) scVI - count data
#Factors: 30
#Factors: 10
F) Zinbwave - count data
#Factors: 30
#Factors: 10
CELseq2
Dropseq
sc_10X
Covariate level
Factors
G) cNMF - count data
#Factors: 30
#Factors: 10
H) scCoGAPs – count data

Supplementary Figure 3) Factor-covariate association heatmaps for benchmarked methods on the scMixology dataset. Factor-covariate association tables for each method: A) sciRED, B) PCA, C) ICA, D) NMF, E) scVI, F) Zinbwave G) cNMF, H) scCoGAPs, and with initial factors set to 30 and 10. Notably, some methods exhibit higher sensitivity to the selected number of factors. For example, ICA and NMF reveal distinct splitting of cell identities for H2228 and H1975, respectively, when the factor number is set to a higher value (K=30). Highlighted example factors are marked with yellow boxes.

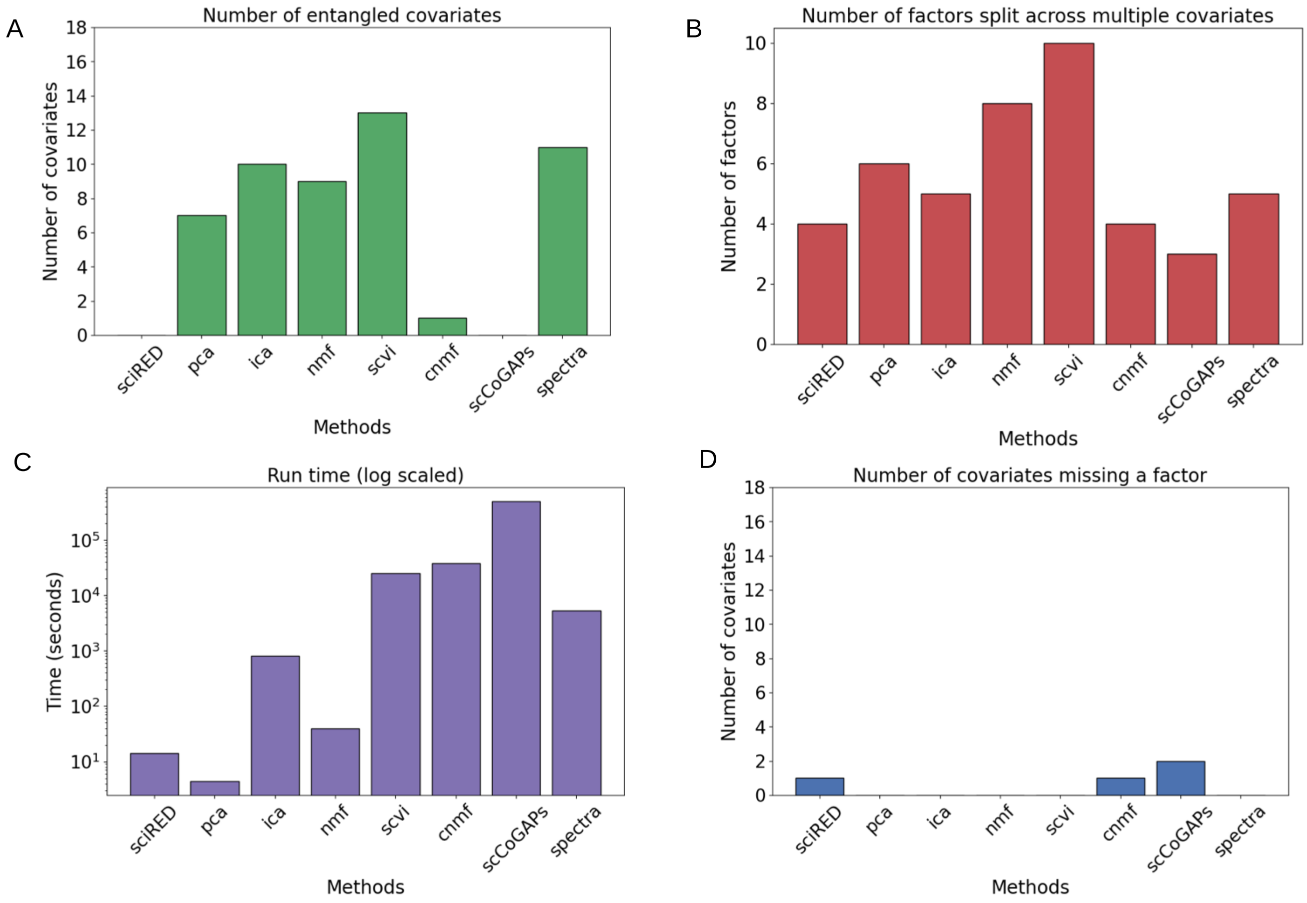

Supplementary Figure 4) Benchmarking sciRED's factor discovery step on a biological dataset. We applied sciRED alongside seven other factor analysis methods (PCA, ICA, NMF, scVI, cNMF, scCoGAPs, Spectra) to a stimulated PBMC dataset to assess factor discovery performance in real single-cell data. Four metrics were defined to compare sciRED's factor discovery with other methods: A) number of entangled covariates, representing the count of covariates associated with multiple factors; B) number of factors split across multiple covariates; C) runtime; and D) number of covariate levels lacking a matching factor. Lower values in all metrics are preferable. Analysis has been performed based on the initial number of factors set to 30 for all methods. scCoGAPs algorithm internally refines the input K (number of patterns) based on the given dataset. sciRED outperformed other methods, with the exception of scCoGAPs, on minimizing entangled covariates and factors split across multiple covariates. The log-scaled runtime plot demonstrates sciRED's scalability, with it outperforming all methods except PCA in terms of runtime efficiency. Notably, scCoGAPs showed poor performance in runtime, requiring over 139 hours to process this dataset. For the number of missed factors, sciRED and cNMF each missed a single technical covariate (sciRED: sample #101; cNMF: sample #1256), while scCoGAPs missed one biological and one technical covariates (Megakaryocytes and sample #1244).

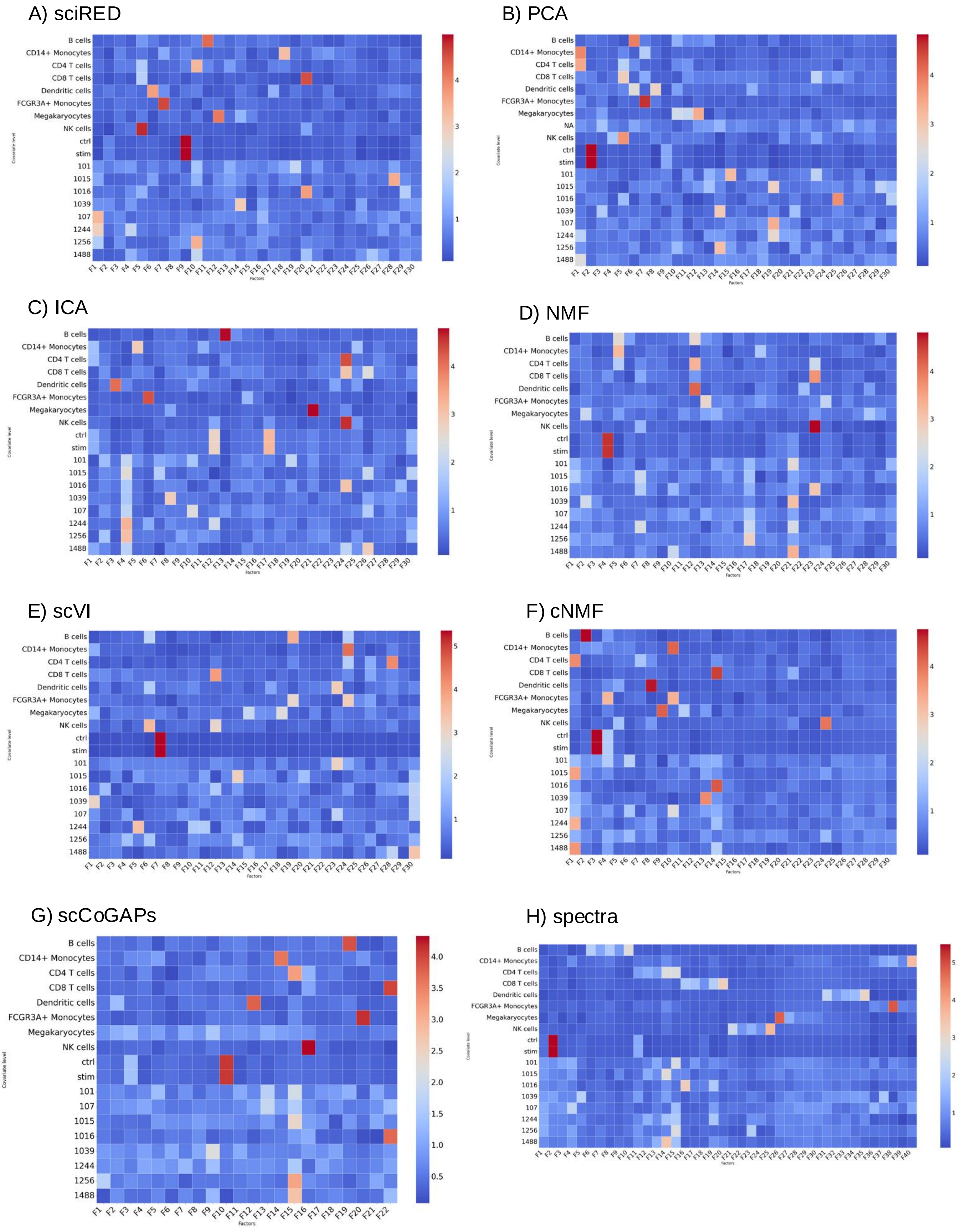
A) sciRED
B) PCA
C) ICA
D) NMF
E) scVI
F) cNMF
G) scCoGAPs
H) spectra
B cells
CD14+ Monocytes
CD4 T cells
CD8 T cells
Dendritic cells
FCGR3A+ Monocytes
Megakaryocytes
NA
NK cells
ctrl
stim
Covariate level
Factors

Supplementary Figure 5) Factor-covariate association heatmaps for benchmarked methods on the stimulated PBMC dataset. Factor-covariate association tables for each method: A) sciRED, B) PCA, C) ICA, D) NMF, E) scVI, F) cNMF, G) scCoGAPs, and H) Spectra.

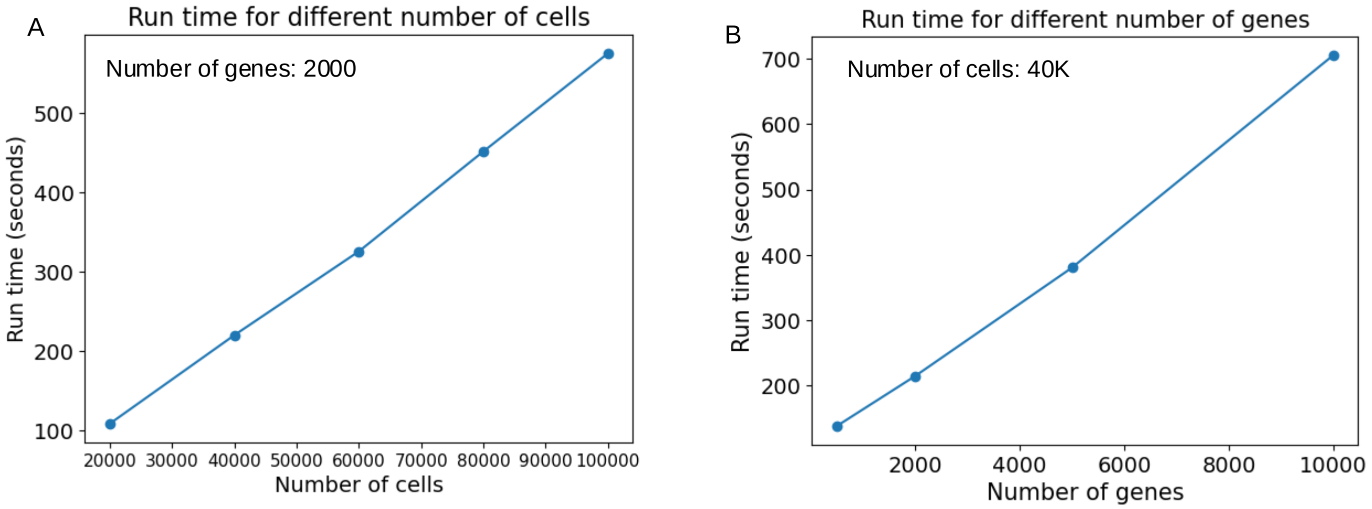

Supplementary Figure 6) Runtime analysis relative to the number of cells and genes. To assess sciRED's runtime scalability, we performed subsampling on the Human Lung Transplants dataset (over 108,000 cells) by varying both cell and gene counts. A) Runtime analysis across different cell counts with the number of genes fixed at 2000. The x-axis shows cell counts of 100K, 80K, 60K, 40K, and 20K. B) Runtime analysis across different numbers of highly variable genes, with the number of cells fixed at 40K. The x-axis shows gene counts of 500, 2000, and 5000. The y-axis on both scatter plots indicates runtime in seconds. Results indicate that sciRED's runtime scales linearly with both the number of cells and genes.

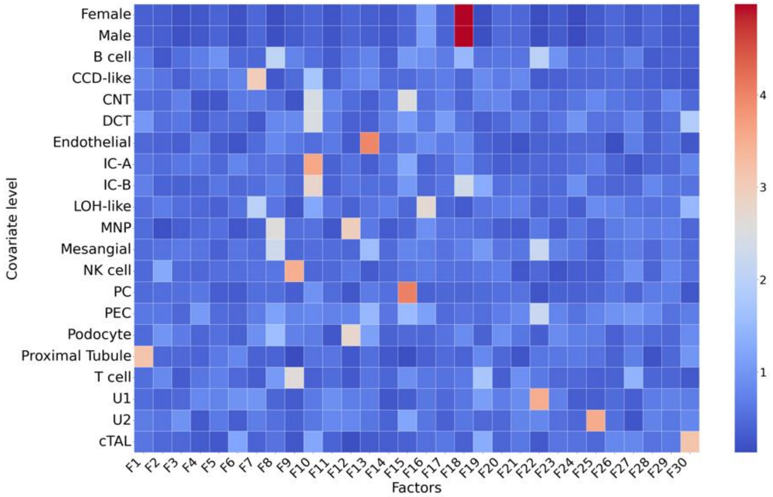

Supplementary Figure 7) The complete factor-covariate association table of the healthy kidney atlas. Association scores between all 30 factors and the sex and cell type covariate levels are shown. sciRED provides both the complete table and a filtered table, which includes factors with at least one association score exceeding the threshold defined over the entire distribution.

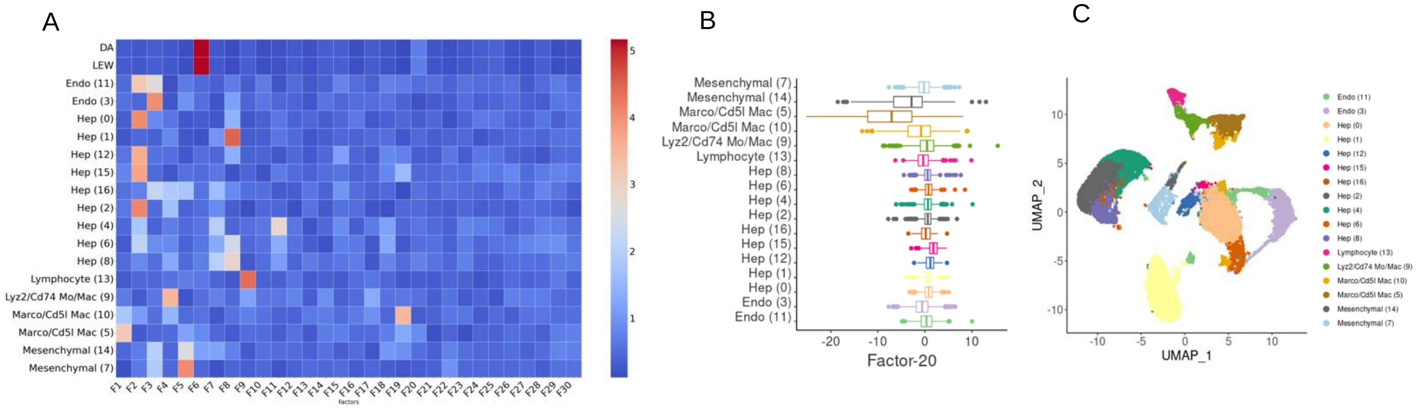

Supplementary Figure 8) Annotated healthy rat liver map. A) Association scores between all 30 factors and the strain and cell type covariate levels are shown. B) Boxplot depicting the distribution of factor F20 across different cell types. The box represents the interquartile range (IQR), with the line indicating the median. Whiskers extend to 1.5 × IQR, and dots denote individual cell factor scores identified as outliers. C) UMAP visualization of annotated healthy rat liver map, each dot represents a cell, with colors indicating cell types.

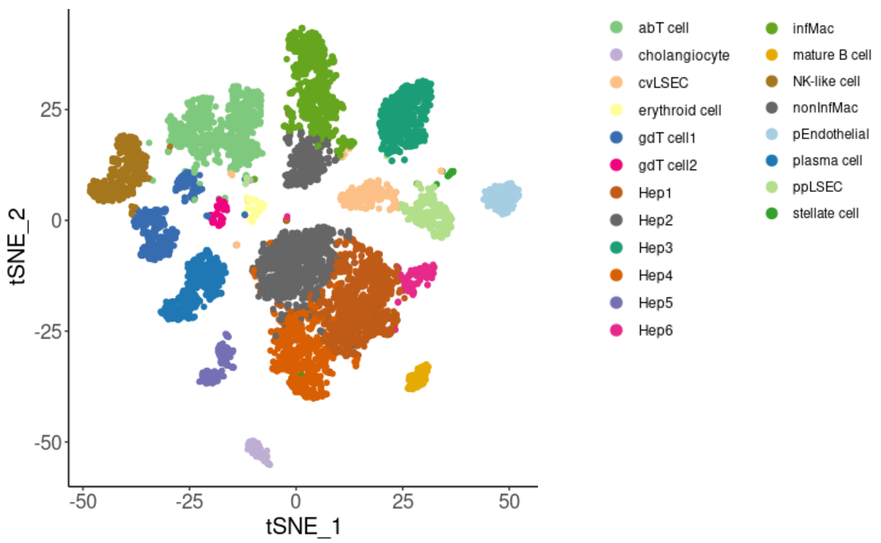

Supplementary Figure 9) Annotated healthy human liver map. t-SNE distribution of annotated healthy human liver map, each dot represents a cell, with colors indicating cell types.

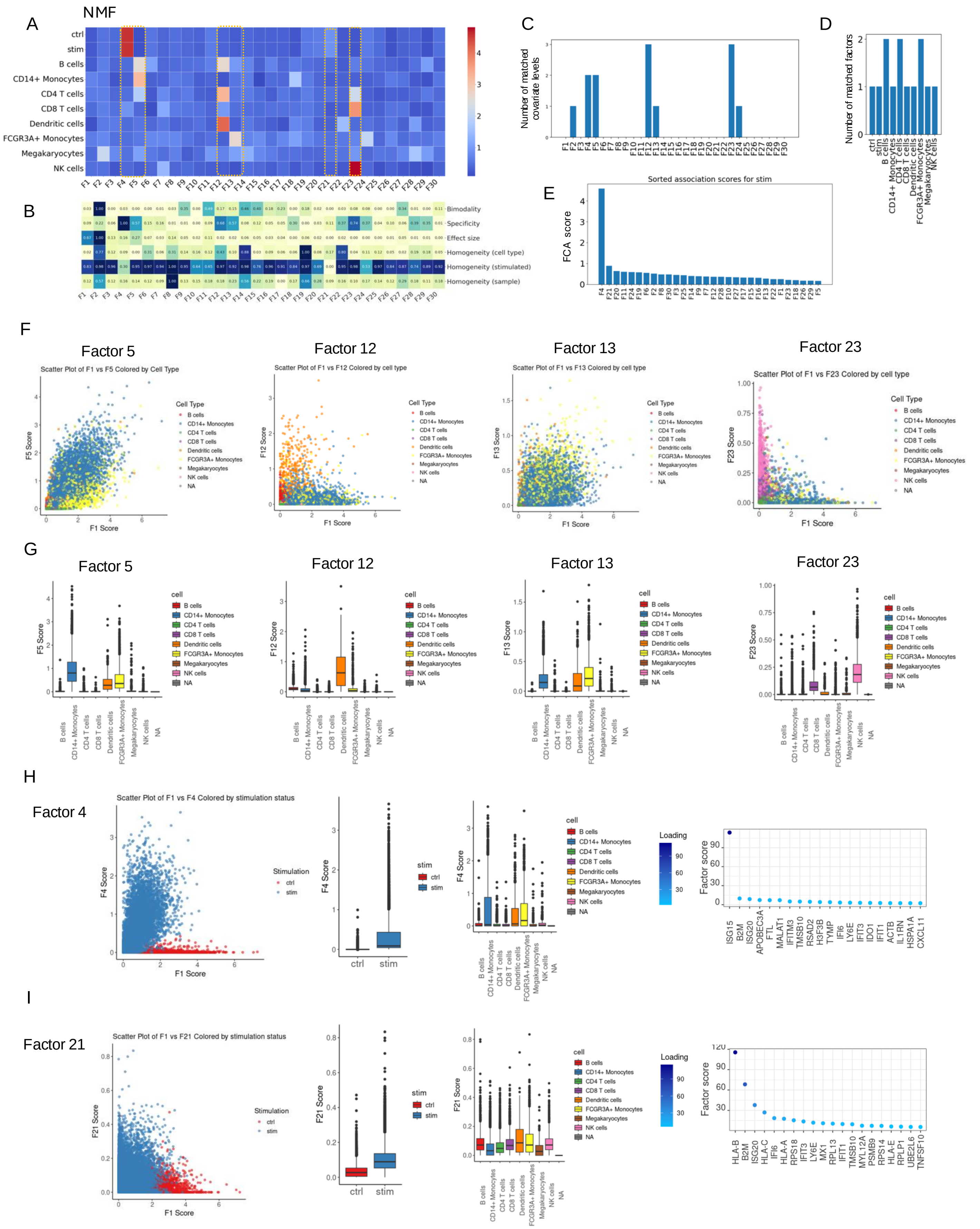

A
NMF
ctrl
stim
B cells
CD14+ Monocytes
CD4 T cells
CD8 T cells
Dendritic cells
FCGR3A+ Monocytes
Megakaryocytes
NK cells
B
Bimodality
Specificity
Effect size
Homogeneity (cell type)
Homogeneity (stimulated)
Homogeneity (sample)
C
Number of matched covariate levels
D
Number of matched factors
E
Sorted association scores for stim
FCA score
F
Factor 5
Scatter Plot of F1 vs F5 Colored by Cell Type
Factor 12
Scatter Plot of F1 vs F12 Colored by cell type
Factor 13
Scatter Plot of F1 vs F13 Colored by cell type
Factor 23
Scatter Plot of F1 vs F23 Colored by cell type
Cell Type
F1 Score
G
Factor 5
Factor 12
Factor 13
Factor 23
cell
NA
H
Factor 4
Scatter Plot of F1 vs F4 Colored by stimulation status
Stimulation
F4 Score
Loading
Factor score
ISG15
B2M
ISG20
APOBEC3A
FTL
MALAT1
IFITM3
TMSB10
RSAD2
H3F3B
TYMP
IFI6
LY6E
IFIT3
IDO1
IFIT1
ACTB
IL1RN
HSPA1A
CXCL11
I
Factor 21
Scatter Plot of F1 vs F21 Colored by stimulation status
F21 Score
HLA-B
B2M
ISG20
HLA-C
IFI6
HLA-A
RPS18
IFIT3
LY6E
MX1
RPL13
IFIT1
TMSB10
MYL12A
PSMB9
RPS14
HLA-E
RPLP1
UBE2L6
TNFSF10

Supplementary Figure 10) Applying sciRED's interpretation and evaluation steps to alternative factor identification methods. To assess the utility of sciRED's factor interpretation and evaluation steps on alternative factor analysis methods to PCA, we substituted sciRED's factor discovery step with NMF and applied the modified pipeline to the PBMC dataset. The resulting factors were evaluated using sciRED's standard interpretation framework. A) The FCA table shows that NMF effectively captures various cell-type identity programs and stimulation signals. However, unlike factors identified by sciRED, NMF factors often associate with multiple covariates, complicating interpretation. Six example factors are highlighted in orange boxes. B) FIS metrics were applied as in the standard pipeline. These metrics can be informative about interpretability of factors of interest. For instance, high bimodality and effect size scores for Factor 2, which is associated with megakaryocytes, highlights it as a promising factor for further evaluation. C–D) Metrics like the number of matched covariate levels and factors per covariate are evaluated similarly to the original sciRED pipeline. E) Best-matching factors for specific covariates, such as simulation status, are identified. F) Scatter plots for Factors F5, F12, F13, and F23 illustrate how these factors capture cell-type identity, with points colored by cell type labels. G) Boxplots for these factors show score distributions across various cell types. F4 and F21 have strong associations with stimulation status, as indicated by FCA heatmap and H–I) scatter plots of cells along F1 vs. F4 and F21, colored by stimulation status, with boxplots comparing factor scores between stimulated and control groups, as well as across cell types. Gene loading score analysis reveals that top-scoring genes in these factors include immune response-related genes, further validating their relevance.

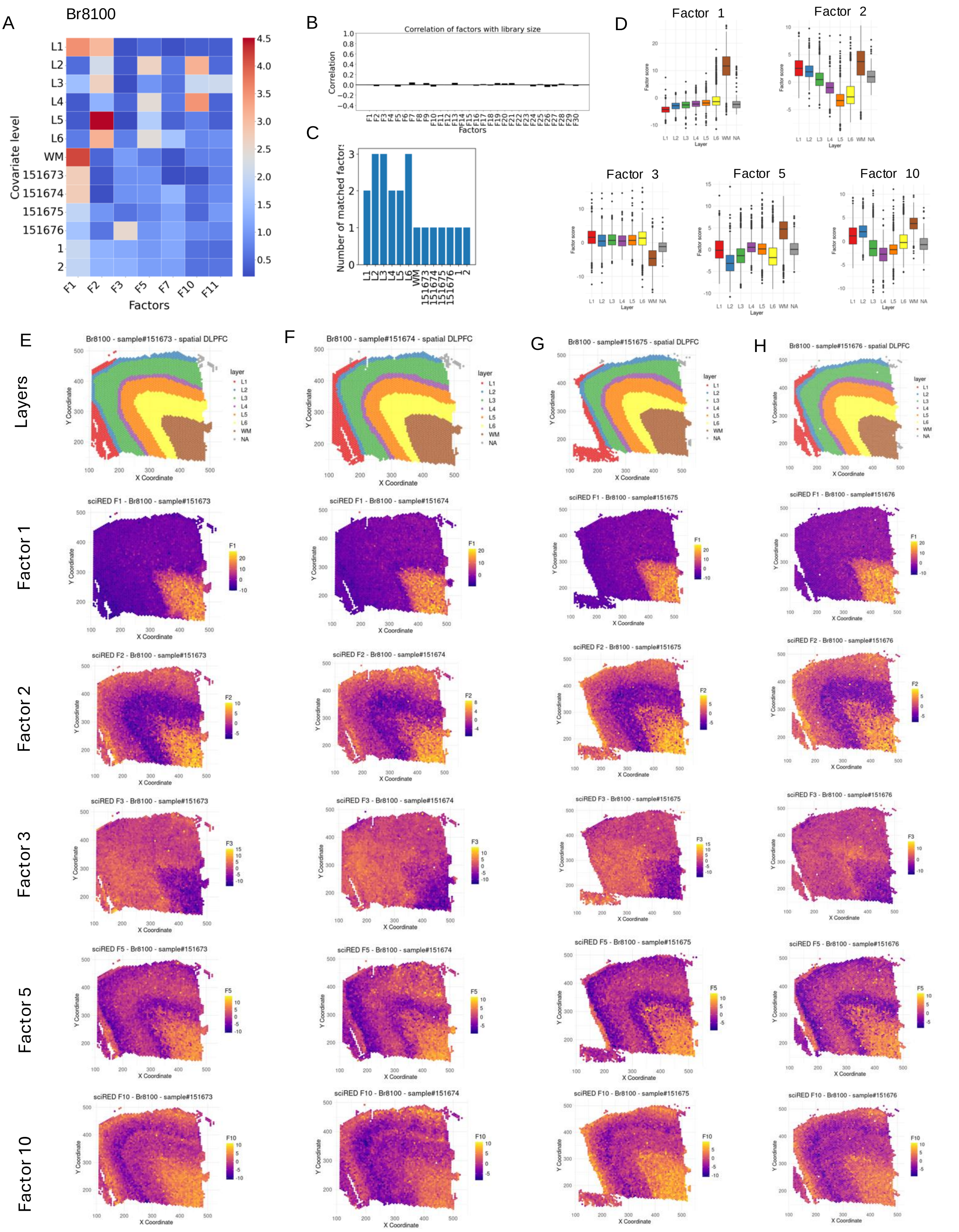
A
Br8100
Covariate level
L1
L2
L3
L4
L5
L6
WM
151673
151674
151675
151676
1
2
Factors
F1
F2
F3
F5
F7
F10
F11
B
Correlation of factors with library size
Correlation
Factors
C
Number of matched factors
D
Factor 1
Factor 2
Factor 3
Factor 5
Factor 10
Factor score
Layer
E
F
G
H
Br8100 - sample#151673 - spatial DLPFC
Br8100 - sample#151674 - spatial DLPFC
Br8100 - sample#151675 - spatial DLPFC
Br8100 - sample#151676 - spatial DLPFC
layer
NA
X Coordinate
Y Coordinate
Layers
Factor 1
Factor 2
Factor 3
Factor 5
Factor 10
sciRED F1 - Br8100 - sample#151673
sciRED F1 - Br8100 - sample#151674
sciRED F1 - Br8100 - sample#151675
sciRED F1 - Br8100 - sample#151676
sciRED F2 - Br8100 - sample#151673
sciRED F2 - Br8100 - sample#151674
sciRED F2 - Br8100 - sample#151675
sciRED F2 - Br8100 - sample#151676
sciRED F3 - Br8100 - sample#151673
sciRED F3 - Br8100 - sample#151674
sciRED F3 - Br8100 - sample#151675
sciRED F3 - Br8100 - sample#151676
sciRED F5 - Br8100 - sample#151673
sciRED F5 - Br8100 - sample#151674
sciRED F5 - Br8100 - sample#151675
sciRED F5 - Br8100 - sample#151676
sciRED F10 - Br8100 - sample#151673
sciRED F10 - Br8100 - sample#151674
sciRED F10 - Br8100 - sample#151675
sciRED F10 - Br8100 - sample#151676

Supplementary Figure 11) Application of sciRED to spatial transcriptomics data (subject Br8100). sciRED was applied to spatial transcriptomics data from Maynard et al. (2020), focusing on the six-layered human dorsolateral prefrontal cortex (DLPFC) to identify spatial gene expression patterns. This study used the 10x Genomics Visium platform to map the spatial organization of gene expression across the six cortical layers and white matter (WM). Each tissue block of DLPFC spans the six cortical layers and WM. Two pairs of spatial replicates were sampled from neurotypical adult donors. Each pair consisted of two directly adjacent, 10-μm serial tissue sections, with the second pair located 300 μm posterior to the first. Here, we applied sciRED to Visium samples from subject Br8100, each consisting of four total samples. Library size was adjusted using sciRED's GLM Poisson, and factor decomposition was performed on the resulting residuals. A) The FCAT heatmap shows associations between factors and cortical layers, sample IDs, and replicate covariates, with Factors 1, 2, 3, 5, and 10 capturing distinct signatures related to cortical layers and WM. B) Low correlation between factors and library size indicates these factors are minimally affected by technical variation related to read count. C) Barplot showing the number of matched factors for each covariate level. D) Boxplot distributions for Factors 1, 2, 3, 5, and 10. The boxes represent the interquartile range (IQR), with the line indicating the median. Whiskers extend to 1.5 × IQR, and dots denote factor scores identified as outliers. E-H) Projections of factor scores across each sample slide, illustrate spatial distribution. For example, Factor 1 captures a WM gene expression signature consistently across all samples, supported by its boxplot distribution and its enrichment in WM regions in each Visium slide.

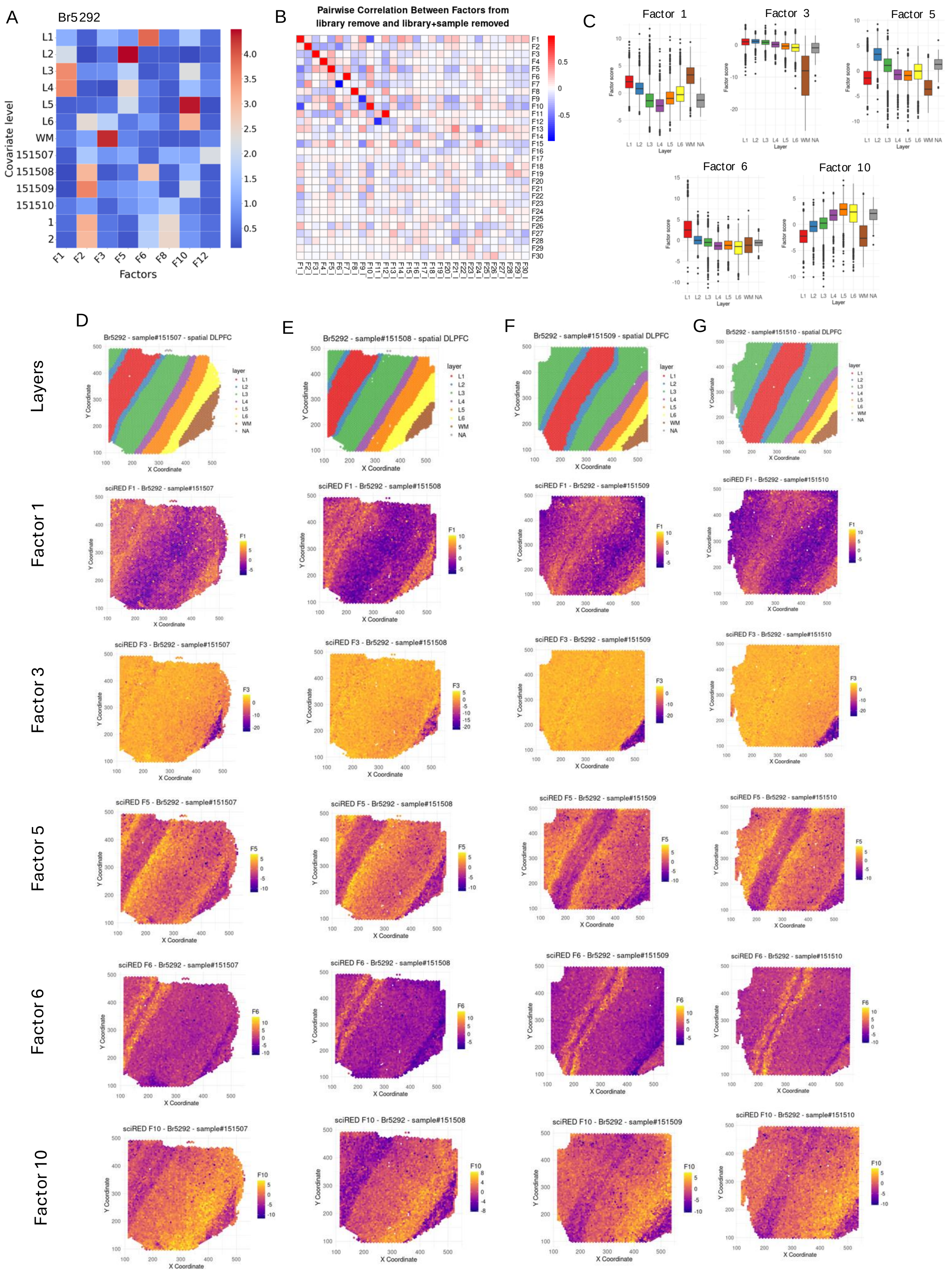

A
Br5292
Covariate level
Factors
B
Pairwise Correlation Between Factors from library remove and library+sample removed
C
Factor 1
Factor 3
Factor 5
Factor 6
Factor 10
Factor score
Layer
D
E
F
G
Layers
Factor 1
Factor 3
Factor 5
Factor 6
Factor 10
Br5292 - sample#151507 - spatial DLPFC
Br5292 - sample#151508 - spatial DLPFC
Br5292 - sample#151509 - spatial DLPFC
Br5292 - sample#151510 - spatial DLPFC
X Coordinate
Y Coordinate
sciRED F1 - Br5292 - sample#151507
sciRED F3 - Br5292 - sample#151507
sciRED F5 - Br5292 - sample#151507
sciRED F6 - Br5292 - sample#151507
sciRED F10 - Br5292 - sample#151507

Supplementary Figure 12) Application of sciRED to spatial transcriptomics data (subject Br5292). Similar to Figure S11, sciRED was applied to spatial transcriptomics samples from another subject, Br5292, demonstrating its effectiveness in capturing dorsolateral prefrontal cortex layer-specific gene expression signatures. A) The FCAT heatmap shows associations between factors, cortical layers, sample IDs, and replicate covariates. B) To assess the impact of adjusting for library size alone versus adjusting for both library size and sample IDs using a Poisson GLM, we conducted a correlation analysis, showing that both approaches yielded equivalent factors. Factors 1, 3, 5, 6, and 10 capture distinct spatial gene expression patterns, as shown by C) boxplot distributions across cortical layers and D-G) the projection of scores across the Visium slides.

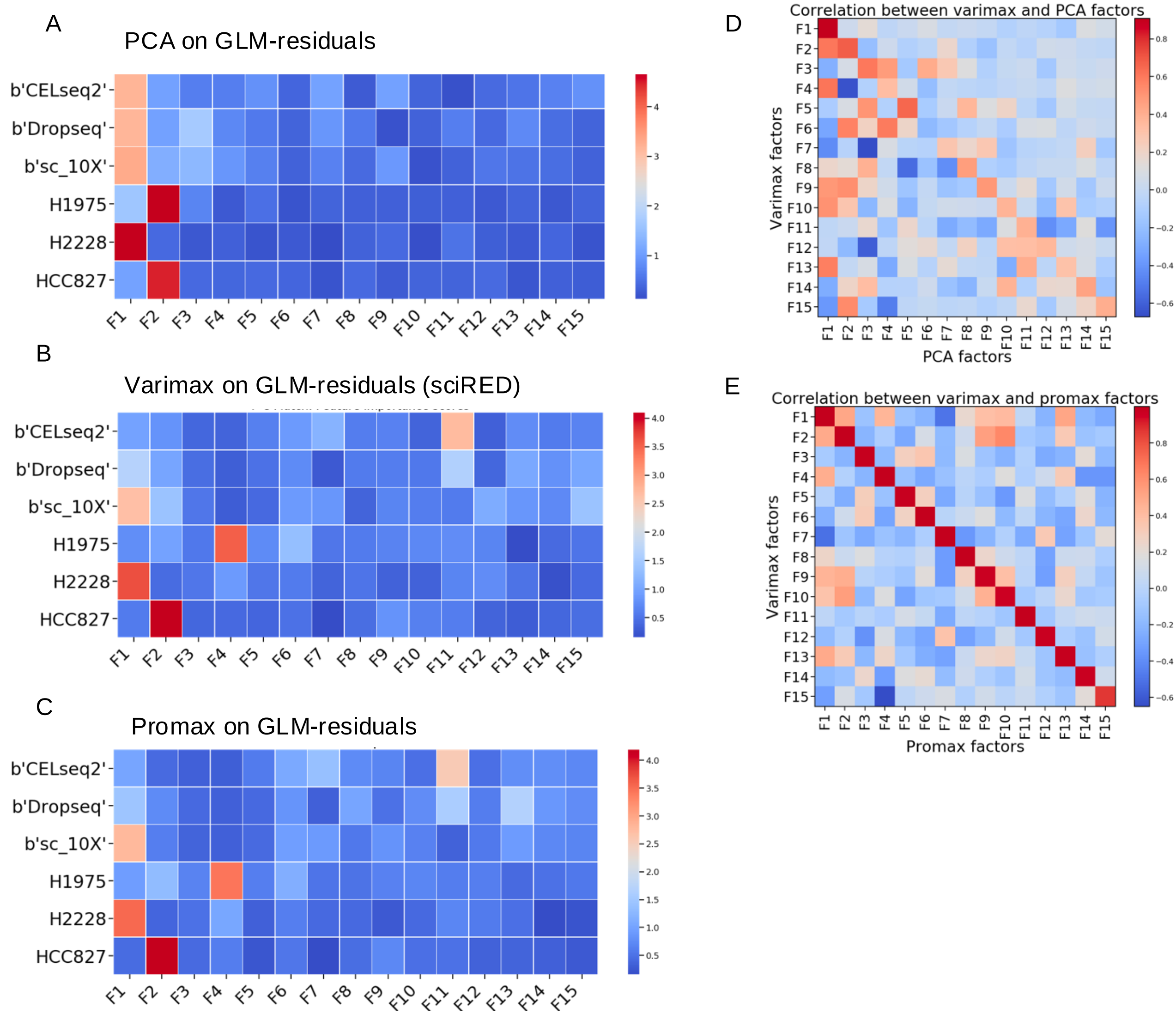

Supplementary Figure 13) Enhancement in identifying cell line-specific factors through rotations. FCA tables depict A) PCA, B) sciRED, and C) Promax-rotated PCs obtained from the Pearson residual of the scMixology dataset after regressing protocol and library size covariates. D) Correlation heatmap illustrates the relationship between varimax and PCs. E) Correlation heatmap between sciRED (varimax-rotated) and promax factors.

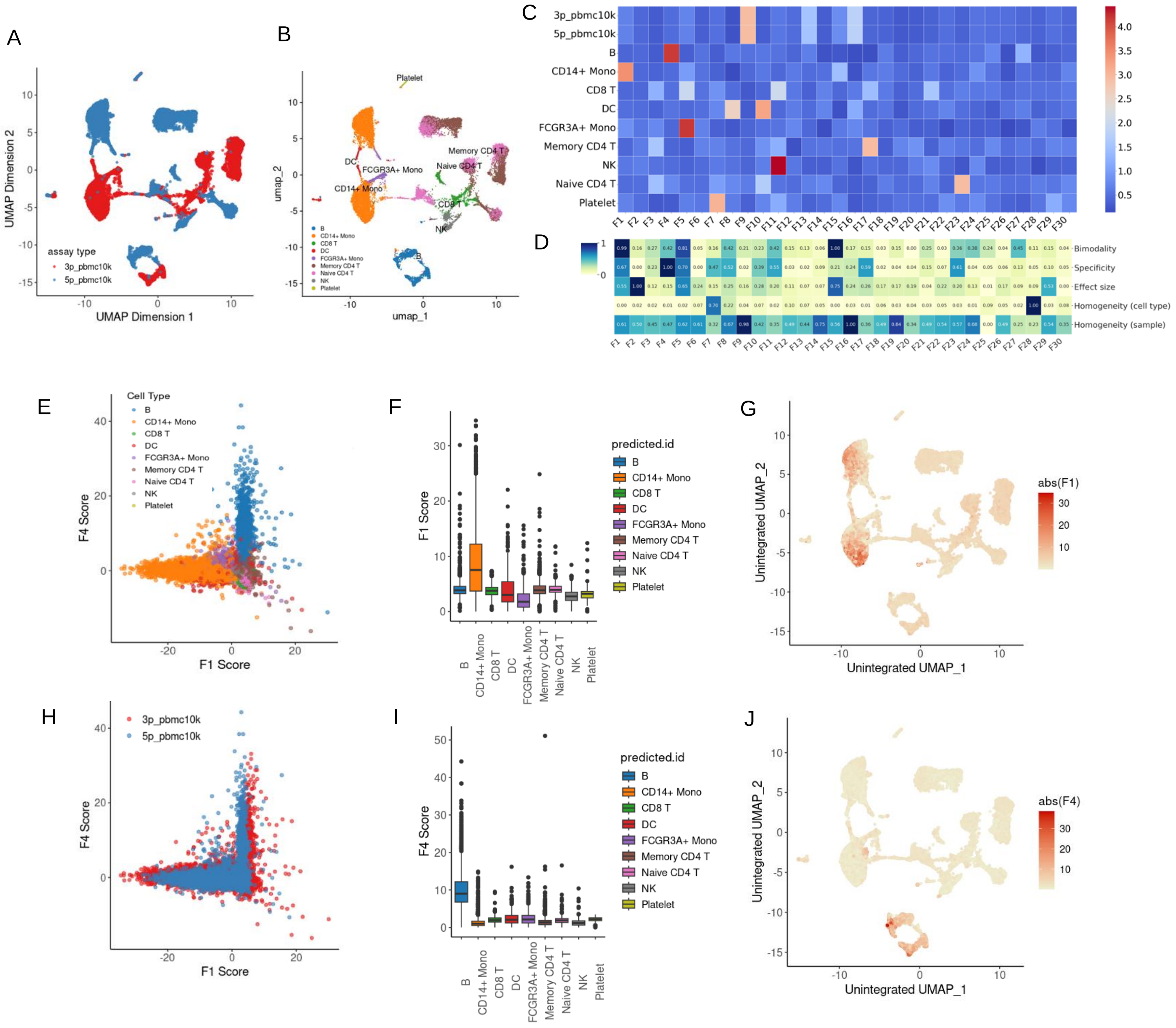

Supplementary Figure 14) sciRED performance in the presence of strong batch effects. To assess sciRED's robustness against batch effects, we applied it to two PBMC datasets profiled with 10x Genomics single-cell 3' and 5' gene expression libraries, where batch effects between assays are substantial. sciRED was run on the combined count matrices, regressing out library and sample IDs in the Poisson GLM step. A) UMAP projection of the merged datasets colored by assay type, and B) UMAP projection colored by cell type labels, both reveal significant batch effects, with poor cell integration observed without correction. C) FCA and D) FIS heatmaps, with examples of factors F1 and F4. E) Scatter plot showing the distribution of cells along F1 and F4, colored by cell type. F) Boxplot of F1 scores across cell type labels. G) Projection of absolute F1 values over the unintegrated UMAP, highlighting its enrichment in CD14+ monocyte clusters from both assays. H) Scatter plot of cell distribution across F1 and F4, colored by assay type. I) Boxplot of F4 scores across cell types. J) Projection of absolute F4 values over the unintegrated UMAP, showing enrichment in B cell clusters across both assays.

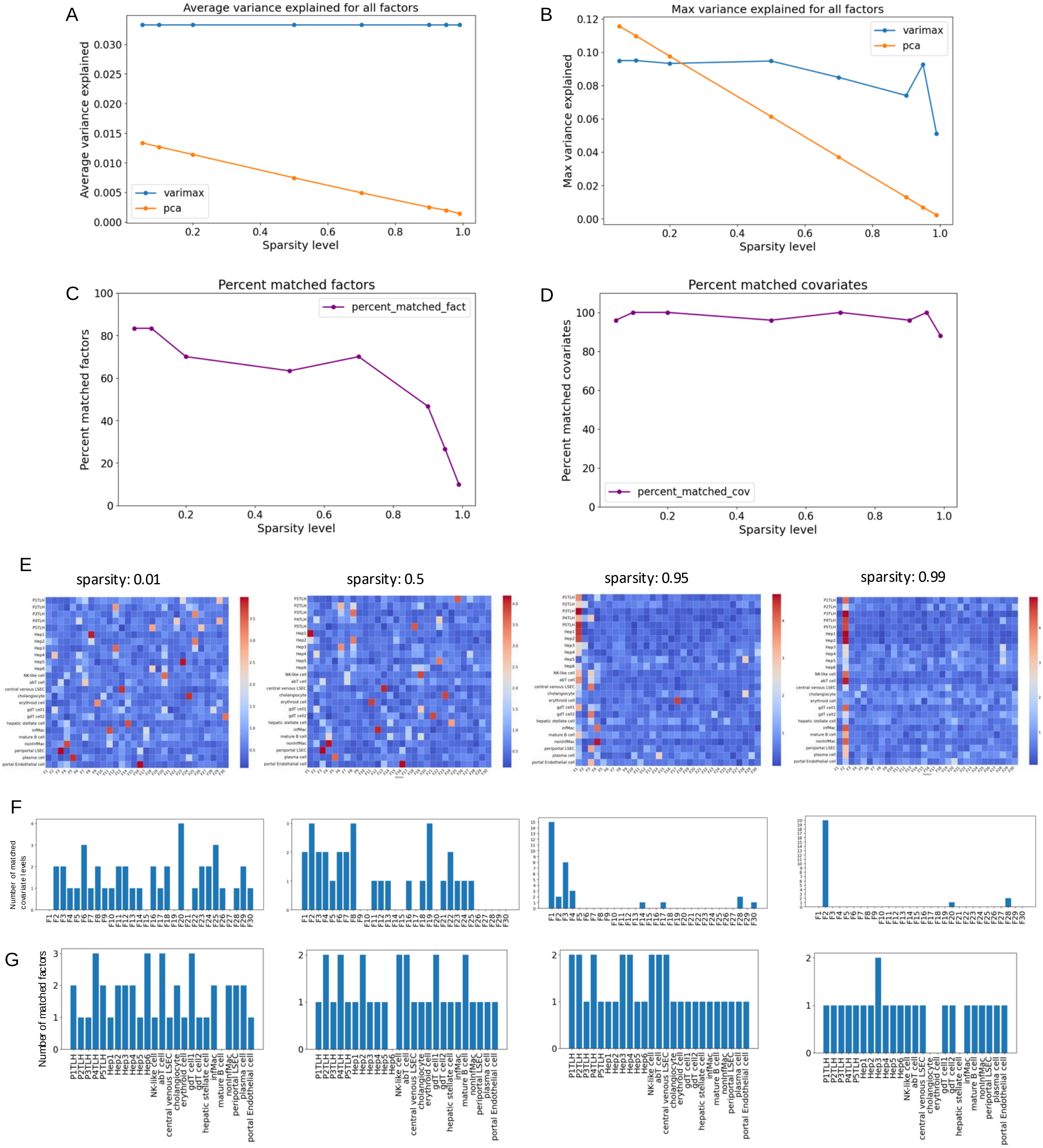

A
Average variance explained for all factors
Average variance explained
Sparsity level
varimax
pca
B
Max variance explained for all factors
Max variance explained
Sparsity level
varimax
pca
C
Percent matched factors
Percent matched factors
Sparsity level
percent_matched_fact
D
Percent matched covariates
Percent matched covariates
Sparsity level
percent_matched_cov
E
sparsity: 0.01
sparsity: 0.5
sparsity: 0.95
sparsity: 0.99
F
Number of matched covariate levels
G
Number of matched factors

Supplementary Figure 15) Evaluation of sparsity effect on sciRED's decomposition results. To assess the impact of data sparsity on sciRED's performance, we varied the sparsity levels (0.01, 0.3, 0.5, 0.7, 0.9, 0.95, 0.99) in the human liver atlas dataset by progressively replacing proportions of gene expression values with zeros. The resulting factors were evaluated based on A) average variance explained across all factors, B) maximum variance explained among factors, C) percentage of matched factors, and D) percentage of matched covariates at each sparsity level. E) FCA heatmaps display factor associations for sparsity levels of 0.01, 0.5, 0.95, and 0.99 as examples. F and G show corresponding bar plots of the number of matched covariate levels per factor and the number of matched factors per covariate level, respectively.

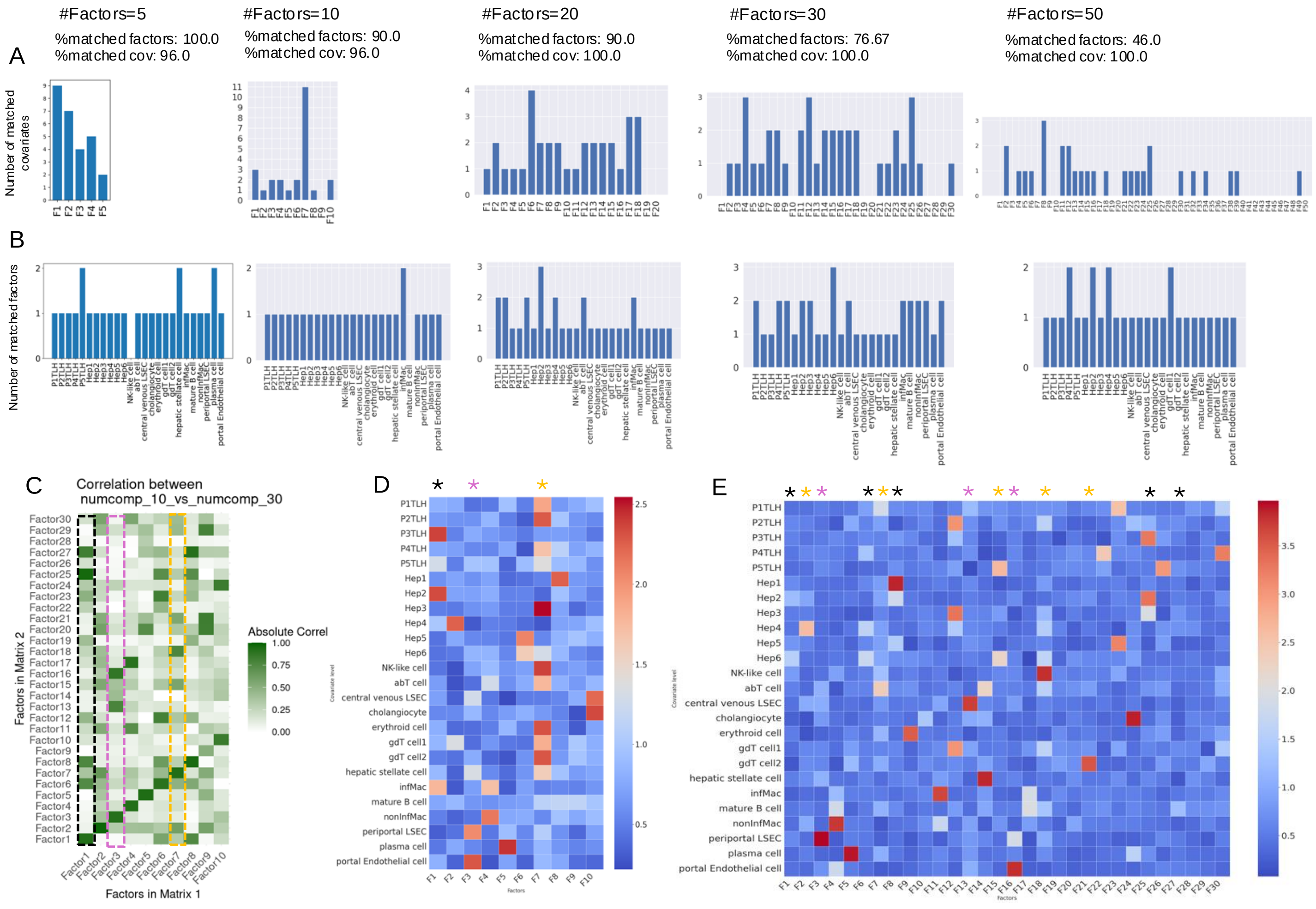

Supplementary Figure 16) Evaluating the effect of factor number on sciRED's decomposition results based on the human liver map. To investigate the impact of varying factor counts on sciRED's performance, we applied decomposition on the human liver map dataset using factor numbers (K) of 5, 10, 20, 30, and 50, comparing the outcomes. A) Number of matched covariates across different factor counts. B) Number of matched factors per covariate level. C) Correlation heatmap between factors derived from K=10 and K=30 decompositions. D and E show FCA heatmaps for K=10 and K=30, respectively. In the K=10 decomposition, three key factors—F1 (black), F3 (pink), and F7 (yellow)—were identified. For the K=30 decomposition, F1 from K=10 maps to F1, F6, F8, F25, and F27 (black box and asterisks); F3 from K=10 corresponds to F3, F13, and F16 in K=30 (pink box and asterisks); and F7 from K=10 aligns with F2, F7, F15, F18, and F21 in K=30 (yellow box and asterisks). These results indicate that limiting factor numbers may cause individual factors to aggregate multiple gene expression programs, reducing interpretability.

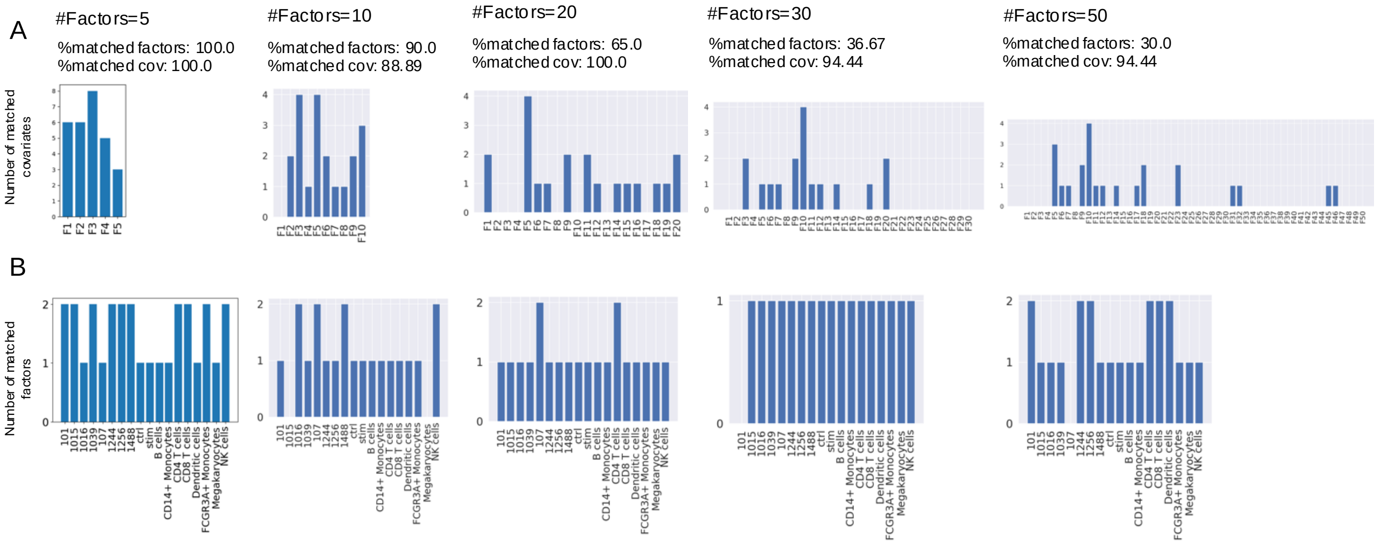

Supplementary Figure 17) Evaluating the effect of factor number on sciRED's decomposition results using the stimulated PBMC dataset. We analyzed the impact of varying factor counts (K) on the decomposition results for the PBMC dataset by applying factor numbers of 5, 10, 20, 30, and 50. A) Number of matched covariates for each factor count. B) Number of matched factors per covariate level. Results were consistent with those from the human liver map: as the factor count increased, each factor tended to align with fewer covariates, and a larger proportion of factors remained unmatched at higher K values.

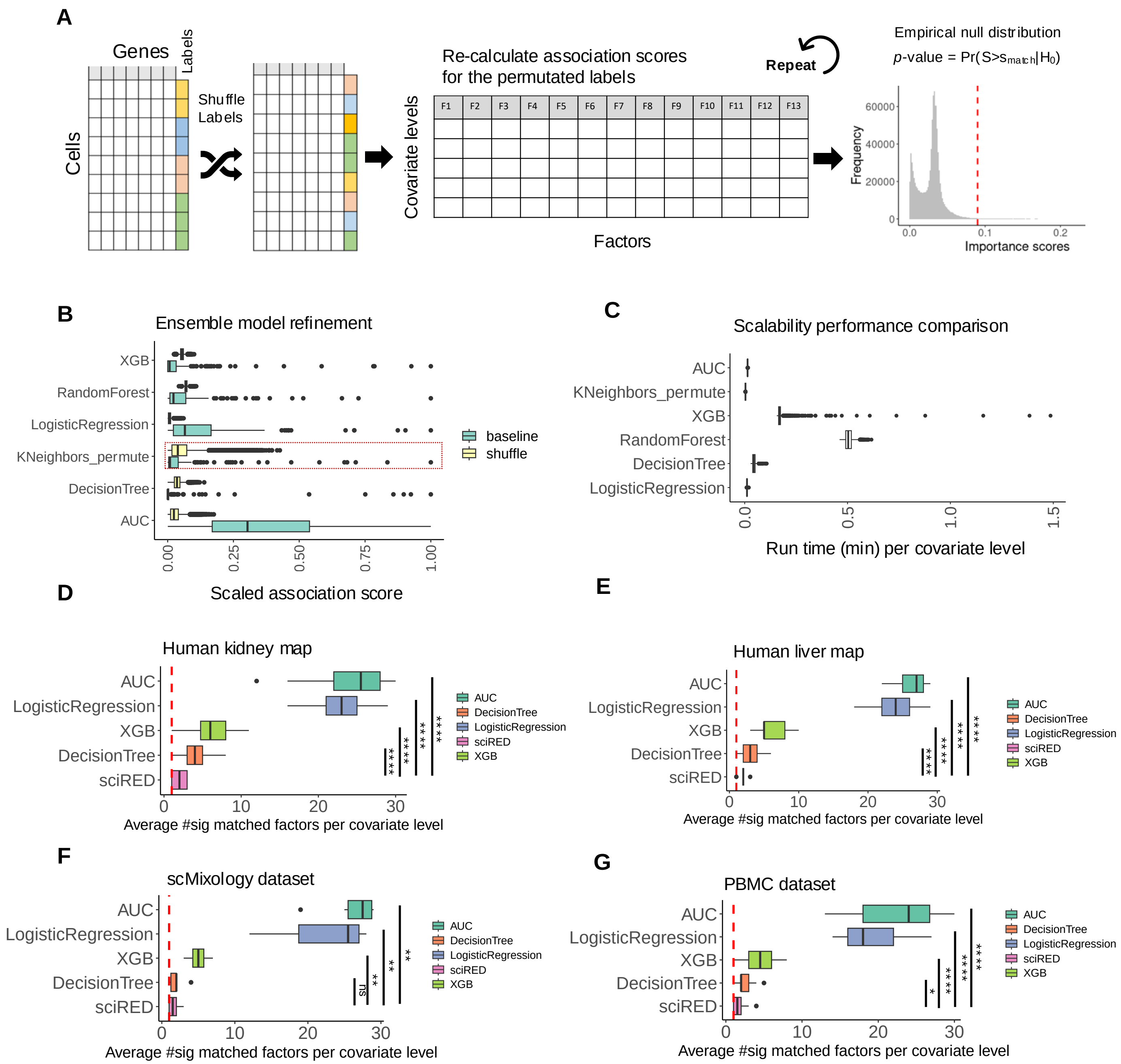
A
Genes
Labels
Cells
Shuffle
Labels
Re-calculate association scores
for the permutated labels
Repeat
Covariate levels
F1
F2
F3
F4
F5
F6
F7
F8
F9
F10
F11
F12
F13
Factors
Empirical null distribution
p-value = Pr(S>s_match|H0)
Frequency
Importance scores
B
Ensemble model refinement
XGB
RandomForest
LogisticRegression
KNeighbors_permute
DecisionTree
AUC
baseline
shuffle
Scaled association score
C
Scalability performance comparison
AUC
KNeighbors_permute
XGB
RandomForest
DecisionTree
LogisticRegression
Run time (min) per covariate level
D
Human kidney map
AUC
LogisticRegression
XGB
DecisionTree
sciRED
Average #sig matched factors per covariate level
E
Human liver map
F
scMixology dataset
G
PBMC dataset

Supplementary Figure 18) Performance evaluation and benchmarking of sciRED. A) Permutation tests were used to assess the significance of each covariate-factor association metric (see methods). B) Comparison of AUC, K-Nearest Neighbors (KNN), logistic regression, decision tree, random forest, and XGB using permutation tests on the scMixology benchmark dataset. We expect the unshuffled (baseline) scores to be higher than the shuffled association scores. KNN's poor performance led to its exclusion from the sciRED ensemble model. The importance scores of each model were min-max scaled for comparison. C) Run time evaluation resulted in the exclusion of the random forest method due to inferior scalability performance. D-G) Benchmark analysis illustrates sciRED's superior performance relative to single classifiers. We assessed the average number of significant matched factors per covariate level for the D) human kidney map, E) human liver map, F) scMixology dataset and G) stimulated PBMC dataset at a significance level of p-value=0.05. The dashed line denotes the value of one factor-covariate association. Model distributions closer to a mean of one and lower variance suggest improved specificity and stability. (p values are based on Wilcoxon test: ns: $p > 0.05$, *: $p <= 0.05$, **: $p <= 0.01$, ***: $p <= 0.001$, ****: $p <= 0.0001$; see Source Data)

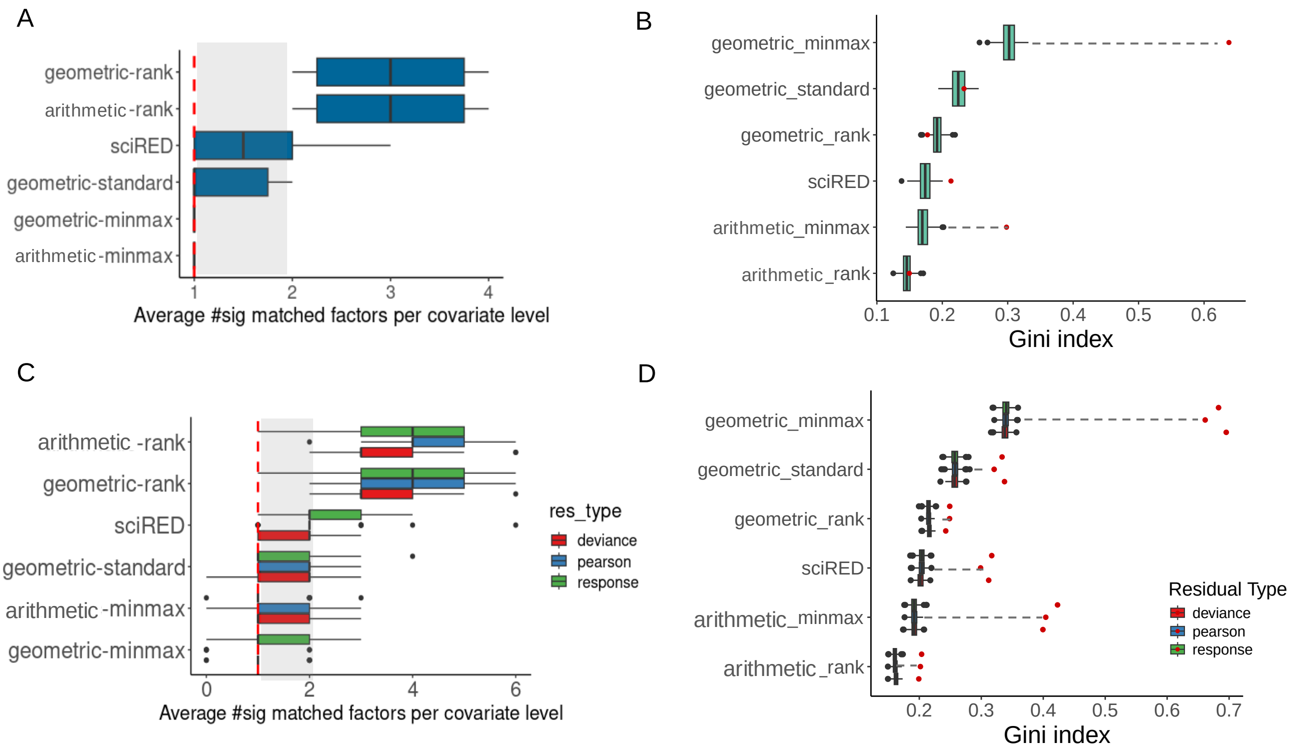

Supplementary Figure 19) Optimizing ensemble design through permutation-based comparison of scaling and mean calculation methods. We compared three scaling methods (standardization, min-max scaling, and rank-based) and two mean calculation approaches (arithmetic and geometric) to design an optimal ensemble. sciRED uses standardization scaling and arithmetic mean calculation, which is the optimal combination based on analysis in this figure (panels A, B are for results based on the scMixology dataset and panels C, D are for results based on the healthy human liver dataset). We used the average number of significant associated factors per covariate (p-value=0.05) along the Gini coefficient (see methods) for comparing ensemble designs. Ideally, the average number of significant associated factors per covariate would be close to one, and the Gini coefficients of the shuffled datasets are expected to be less than for the unshuffled data. A) Average number of significant associated factors per covariate (p-value=0.05). The dashed line indicates the value of one factor-covariate association, while the grey area represents the approximate acceptable range. Distributions closer to a mean of one indicate improved specificity. B) Distribution of Gini coefficient calculated for FCA tables of shuffled datasets for each ensemble table. Red dots represent the Gini coefficient of the unshuffled dataset's FCA table, expected to be higher than for the shuffled data (see methods). C) Box plot representing the average number of significant associated factors per covariate (p-value=0.05) for the healthy human liver dataset based on various residual types. Deviance, response and Pearson residuals show comparable performance with Pearson showing lower variance for sciRED (standardization, arithmetic mean). D) Boxplots indicating the distribution of the Gini coefficient calculated for FCA tables of shuffled datasets for each ensemble model and residual type. Deviance, response, and Pearson residuals show comparable results. This analysis reveals that sciRED exhibits superior performance compared to geometric-standard, geometric-rank, and arithmetic-rank methods, as evidenced by the Gini measure. Furthermore, it outperforms ensembles utilizing min-max (which tend to exhibit high false negatives) and rank-scaled methods (associated with high false positives) when considering the average number of significant factors per covariate. The ensemble model was constructed by standardizing the importance scores of AUC, logistic, XGB, and decision tree, followed by arithmetic mean calculation.

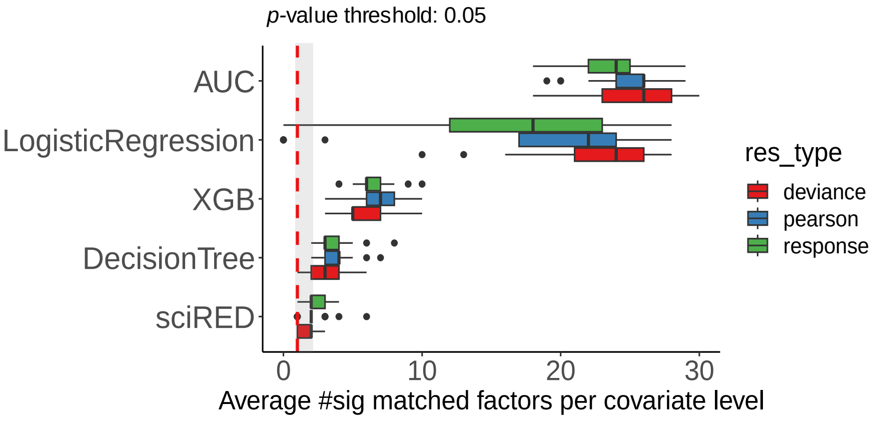

Supplementary Figure 20) Impact of residual choice on factor interpretability. We investigated how various residual-based normalization methods affect downstream analysis, as explored by several studies. Specifically, we examined the impact of different residual types (response, Pearson, and deviance) on factor interpretability performance using the healthy human liver atlas. We employed a permutation test to evaluate the significance of each covariate-factor association based on empirical p-value calculation. Using this approach, we calculated the average number of significant associated factors per covariate (p-value =0.05). Our permutation analysis revealed consistent outcomes across all residual types for most models, with the exception of the response residual-based logistic regression model, which exhibited less stable results. This analysis underscores the robustness of sciRED's performance across different residual types. Consequently, we chose the Pearson residual as the default approach, given its widespread adoption in the field. The dashed line indicates the value of one factor-covariate association, and the grey area represents the approximate acceptable range. The boxes represent the interquartile range (IQR), with the line indicating the median. Whiskers extend to 1.5 × IQR, and dots denote outliers.

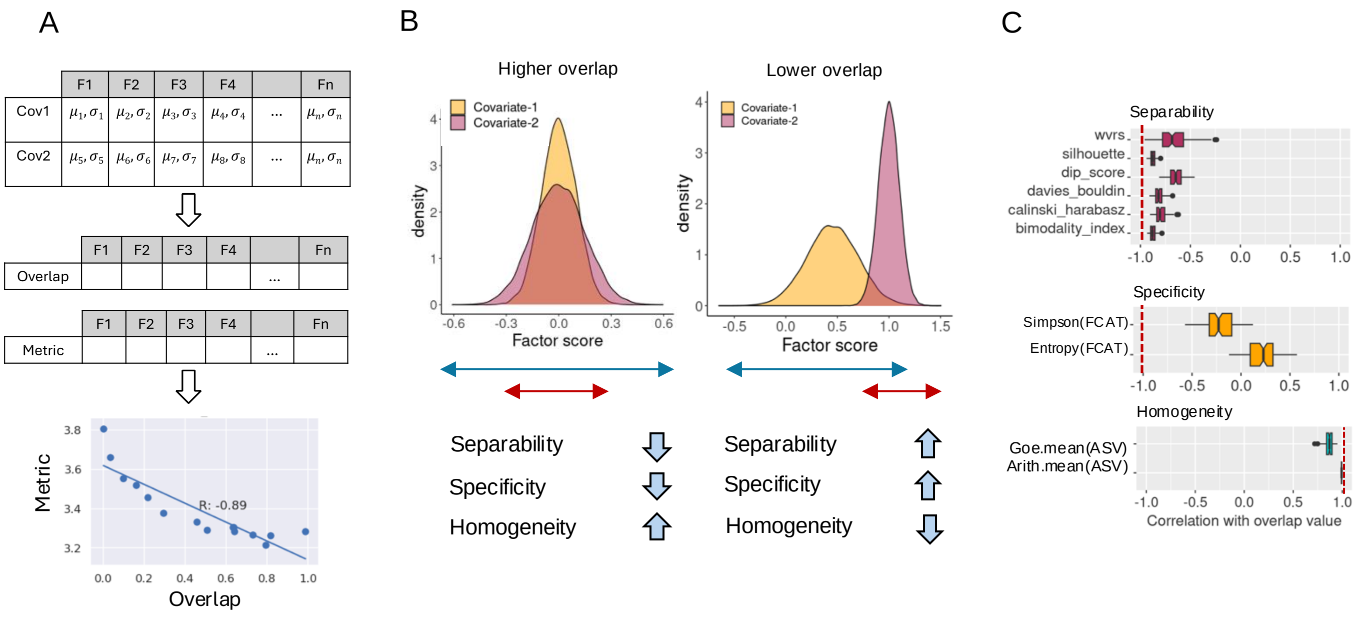

Supplementary Figure 21) Evaluating factor interpretability metrics through factor simulation. Factors were simulated to evaluate the effectiveness of the proposed factor interpretability metrics. A) Simulation assumes that factors are generated from a Gaussian mixture distribution, where each Gaussian represents cells associated with a covariate level. Correlation between the overlap of the two Gaussian distributions and the metric value was used to assess interpretability. B) Factors with greater overlap values would exhibit lower separability and specificity scores (negative correlation), along with higher homogeneity values (positive correlation), and vice versa. C) Distribution of correlation scores for each metric across various categories. The ideal correlation value is represented by a dashed red line for each interpretability category.
