# Supplementary material for: Interpretable single-cell factor decomposition using sciRED
Source: Nat Commun. 2025 Feb 22;16:1878. doi: 10.1038/s41467-025-57157-2 (PMC11846867; doi:10.1038/s41467-025-57157-2)
Supplement: Supplementary file 2 — Reporting Summary [file 41467_2025_57157_MOESM2_ESM.pdf]

nature portfolio

Corresponding author(s): Gary Bader

Last updated by author(s): Jan 3, 2025

Reporting Summary

Nature Portfolio wishes to improve the reproducibility of the work that we publish. This form provides structure for consistency and transparency in reporting. For further information on Nature Portfolio policies, see our Editorial Policies and the Editorial Policy Checklist.

Statistics

For all statistical analyses, confirm that the following items are present in the figure legend, table legend, main text, or Methods section.

n/a	Confirmed	
☐	☒	The exact sample size (*n*) for each experimental group/condition, given as a discrete number and unit of measurement
☐	☒	A statement on whether measurements were taken from distinct samples or whether the same sample was measured repeatedly
☐	☒	The statistical test(s) used AND whether they are one- or two-sided<br>*Only common tests should be described solely by name; describe more complex techniques in the Methods section.*
☐	☒	A description of all covariates tested
☐	☒	A description of any assumptions or corrections, such as tests of normality and adjustment for multiple comparisons
☐	☒	A full description of the statistical parameters including central tendency (e.g. means) or other basic estimates (e.g. regression coefficient) AND variation (e.g. standard deviation) or associated estimates of uncertainty (e.g. confidence intervals)
☐	☒	For null hypothesis testing, the test statistic (e.g. *F*, *t*, *r*) with confidence intervals, effect sizes, degrees of freedom and *P* value noted<br>*Give P values as exact values whenever suitable.*
☒	☐	For Bayesian analysis, information on the choice of priors and Markov chain Monte Carlo settings
☒	☐	For hierarchical and complex designs, identification of the appropriate level for tests and full reporting of outcomes
☐	☒	Estimates of effect sizes (e.g. Cohen's *d*, Pearson's *r*), indicating how they were calculated

Our web collection on statistics for biologists contains articles on many of the points above.

Software and code

Policy information about availability of computer code

Data collection	NA
Data analysis	The Python package for sciRED, along with example case scenarios and the analysis code used in this manuscript, is freely accessible at https://github.com/BaderLab/sciRED. The code is archived and citable via Zenodo: Pouyabahar et al., sciRED, GitHub repository, DOI: 10.5281/zenodo.14593137 (2024).<br><br>R packages:<br>SoupX (version: 1.6.2)<br>ggplot2 (version 3.5.1)<br>gprofiler2 (version 0.2.3)<br><br>Python packages:<br>scikit-learn (version 0.22.1)<br>scipy (version 1.4.1)<br>scikit-image (version 0.23.2)<br>numpy (version 1.26.4)<br>pandas (version 2.2.1)<br>scanpy (version 1.10.1)<br>statsmodels (version 0.11.0)<br>seaborn (version 0.13.2)

umap-learn (version 0.5.6)
matplotlib (version 3.7.3)
xgboost (version 2.0.3)
scikit-image (version 0.22.0)
diptest (version 0.2.0)

For manuscripts utilizing custom algorithms or software that are central to the research but not yet described in published literature, software must be made available to editors and reviewers. We strongly encourage code deposition in a community repository (e.g. GitHub). See the Nature Portfolio guidelines for submitting code & software for further information.

Data

Policy information about availability of data

All manuscripts must include a data availability statement. This statement should provide the following information, where applicable:

- Accession codes, unique identifiers, or web links for publicly available datasets
- A description of any restrictions on data availability
- For clinical datasets or third party data, please ensure that the statement adheres to our policy

No new data were generated for this manuscript. The external sources used as input datasets are fully detailed in the 'Data preprocessing' section of the Methods.

Data Availability
scMixology
The scMixology17 dataset includes three human lung adenocarcinoma cell lines: HCC827, H1975, and H2228 [https://github.com/LuyiTian/sc_mixology]. These cell lines were cultured individually and subsequently processed. Single cells from each cell line were combined in equal proportions and libraries were generated using three protocols: CEL-seq2, Drop-seq, and 10x Genomics Chromium. The processed count data was obtained using the scPipe package in R and converted to .h5ad for import into Python. The data underwent log normalization and standardization using the "StandardScaler" function from scikit-learn package.

Interferon (IFN)-β stimulated PBMC dataset
The stimulated data was downloaded from muscData package (Kang18_8vs8, GEO: GSE96583)26 [https://github.com/HelenaLC/muscData]. This dataset includes 10x Genomics droplet-based scRNA-seq PBMC data from eight lupus patients before and after 6h-treatment with interferon-beta. Count data was extracted, and analyzed using sciRED (number of components(k)=30) while modeling library size as a technical covariate. Three outlier cells were removed from the sciRED cell-by-factor score matrix, and factors F2 and F9 scores for 29,062 cells were visualized in Figure 3.

Healthy human kidney atlas
The healthy human kidney map was constructed based on 19 living donors (10 female, 9 male)28 including the transcriptomes of 27,677 cells. The processed files were downloaded from the UCSC Cell Browser [https://cells.ucsc.edu/?ds=living-donor-kidney]. Filtered and normalized data was downloaded and analyzed using sciRED (k=30).

Healthy rat liver atlas
The healthy rat total liver homogenate map includes four whole livers which were acquired from 8-10 week-old healthy male Dark Agouti and Lewis strain rats, and the resulting total liver homogenates went through two-step collagenase digestion and 10x Genomics droplet-based scRNA-seq29. The processed healthy rat liver total homogenate map was downloaded from the UCSC Cell Browser [https://cells.ucsc.edu/?ds=rat-liver-atlas] (GEO: GSE220075). Five outlier cells were removed from the score matrix, and factors F6 and F20 scores for 23,036 cells were visualized in Figure 4. sciRED (k=30) was applied to the count data while modeling library size as a technical covariate. Sample was not included as technical covariate to preserve the strain-specific variations. SoupX31 software (version: 1.6.2) was used to identify genes with the greatest contribution to the ambient RNA. We used the default automatic contamination fraction estimation (Rho) feature in the SoupX (autoEstCont function) to estimate Rho for each sample included in the total liver homogenate map of healthy rat livers. Subsequently, we extracted the estimated ambient RNA profile and identified the top 50 genes contributing the most to the ambient RNA in each sample. Genes were selected if they ranked among the high-scoring ambient RNA contributors in at least two samples. These selected genes were assessed for their presence among the strain-specific myeloid markers identified using both sciRED and standard differential expression methods. Differential expression analysis between the DA and LEW strains within the myeloid population (cluster 5) of the rat liver map was conducted using Seurat's FindMarkers function with default parameters (logfc.threshold = 0.1, min.pct = 0.01, min.cells.feature = 3, and min.cells.group = 3), implementing the non-parametric Wilcoxon rank-sum test.

Healthy human liver atlas
The healthy human liver map32 includes 8,444 parenchymal and non-parenchymal cells obtained from the fractionation of fresh hepatic tissue from five human livers. The liver tissue was obtained from livers procured from deceased donors deemed acceptable for liver transplantation. Data was downloaded from the R package HumanLiver, available at https://github.com/BaderLab/HumanLiver, and sciRED (k=30) was applied to filtered count while modeling library size as a technical covariate.

Human lung transplant dataset
We downloaded the human lung transplants dataset27 from the cellxgene platform (GEO: GSE220797), [https://www.ncbi.nlm.nih.gov/geo/query/acc.cgi?acc=GSE220797] which includes donor lung biopsies from six transplant cases and over 108,000 cells. We then performed subsampling on both genes and cells to systematically assess sciRED's runtime across different dataset sizes.

PBMC 3' and 5' datasets
The two filtered 10k PBMC datasets, profiled using 10x Genomics single-cell 3' and 5' gene expression libraries, were downloaded directly from the 10x Genomics website (3p: https://cf.10xgenomics.com/samples/cell-exp/4.0.0/Parent_NGSC3_DI_PBMC/Parent_NGSC3_DI_PBMC_filtered_feature_bc_matrix.h5, 5p: https://cf.10xgenomics.com/samples/cell-vdj/5.0.0/sc5p_v2_hs_PBMC_10k/sc5p_v2_hs_PBMC_10k_filtered_feature_bc_matrix.h5).

Spatial transcriptomics data
We applied sciRED to spatial transcriptomics data from Maynard et al. (2020)83, focusing on identifying spatial gene expression patterns within the six-layered human dorsolateral prefrontal cortex (DLPFC). Specifically, we selected Visium samples from two subjects (Br8100 and Br5292), with four samples per subject. The data was downloaded using spatialLIBD::fetch_data() [DOI: 10.18129/B9.bioc.spatialLIBD] and then subset by subject. sciRED's Poisson GLM was applied to adjust

for library size.

The Source Data file supports the analyses and visualizations presented in the manuscript, providing factor loading tables for: the kidney dataset (Figures 2f and 2g), the PBMC dataset (Figures 3f, 3g, 3i, and 3j), the rat liver dataset (Figures 4g and 4h), and the human liver dataset (Figures 5e–5h). It also includes SoupX ambient RNA contamination estimates for each rat liver sample (Figure 4g). Additionally, for supplementary analyses, the file contains sciRED loadings for the scMixology dataset with 30 factors (Figure S3) and for spatial samples of the dorsolateral cortex, specifically samples Br8100 and Br5292 (Figures S11 and S12). The exact p-values in Figure S18 are reported in the Source Data file.

Research involving human participants, their data, or biological material

Policy information about studies with human participants or human data. See also policy information about sex, gender (identity/presentation), and sexual orientation and race, ethnicity and racism.

Reporting on sex and gender	The healthy human kidney map was constructed based on 19 living donors (10 female, 9 male) including the transcriptomes of 27,677 cells. Please refer to the original paper's reporting summary for further information:<br>"Patient sex information was collected and considered in study design. Gender information was not collected. This is reported in Supplementary Table 2, as well as figures 2 and 3" [https://www.nature.com/articles/s41467-022-35297-z#data-availability]
Reporting on race, ethnicity, or other socially relevant groupings	No socially-constructed variable has been used. sciRED removes user-defined unwanted technical factors, such as library size and sample or protocol, as covariates within a Poisson generalized linear model (GLM). This regresses out the covariates and produces Pearson residuals.
Population characteristics	sex-specific variations were evaluated within the healthy human kidney map. Please check the original paper for further information:https://www.nature.com/articles/s41467-022-35297-z#data-availability]
Recruitment	No human sample was directly gathered in this study.
Ethics oversight	No human sample was directly gathered in this study.

Note that full information on the approval of the study protocol must also be provided in the manuscript.

Field-specific reporting

Please select the one below that is the best fit for your research. If you are not sure, read the appropriate sections before making your selection.

☒ Life sciences ☐ Behavioural & social sciences ☐ Ecological, evolutionary & environmental sciences

For a reference copy of the document with all sections, see nature.com/documents/nr-reporting-summary-flat.pdf

Life sciences study design

All studies must disclose on these points even when the disclosure is negative.

Sample size	Interferon (IFN)-β stimulated PBMC dataset:<br>The stimulated data was downloaded from muscData package (Kang18_8vs8, GEO: GSE96583)26 . This dataset includes 10x Genomics droplet-based scRNA-seq PBMC data from eight lupus patients before and after 6h-treatment with interferon-beta.<br>Healthy human kidney atlas:<br>The healthy human kidney map was constructed based on 19 living donors (10 female, 9 male)25 including the transcriptomes of 27,677 cells.<br>Healthy rat liver atlas:<br>The healthy rat total liver homogenate map includes four whole livers which were acquired from 8-10 week-old healthy male Dark Agouti and Lewis strain rats, and the resulting total liver homogenates went through two-step collagenase digestion and 10x droplet-based scRNA-seq
Data exclusions	Interferon (IFN)-β stimulated PBMC dataset: Three outlier cells (out of 29,065 total cells) were removed from the sciRED cell-by-factor score matrix. These cells likely represent low-quality data, and their removal had a negligible impact on the overall analysis. The factor scores for F2 and F9 across the remaining 29,062 cells are visualized in Figure 3<br><br>Healthy rat liver atlas:<br>Five outlier cells (out of 23,041 total cells) were removed from the score matrix, likely representing low-quality data. Their removal had a negligible effect on the analysis, and the factor scores for F6 and F20 across the remaining 23,036 cells are visualized in Figure 4.
Replication	Experimental validation for two of the four datasets (human kidney and rat liver) was conducted by independent studies and published separately. The results obtained for the human liver and PBMC datasets were consistent with findings reported in the literature.<br><br>- sciRED finds cell type identity programs and sex-specific processes in a human healthy kidney map: Consistent with the original study, pathway analysis shows an increase in processes related to aerobic metabolism (such as aerobic respiration, oxidative phosphorylation,

tricarboxylic acid (TCA) cycle, and electron transport chain) in males (Figure 2FG). These findings align with the higher basal respiration and ATP-linked respiration processes in males, as functionally validated in the original study.

- sciRED alleviates ambient RNA contamination for group-based comparison:
These myeloid specific strain variations were experimentally validated in the original study.

No direct sample collection was conducted in this study; instead, we relied on the sample size calculations from the original study design. To ensure signals were distinguishable from sample identity, at least two samples were included for each known covariate under investigation (sex, strain, and stimulation status).

Randomization	No direct sample collection was performed in this study; we relied on the original data study design. To account for technical covariates, sciRED removes user-defined unwanted technical factors, such as library size and sample or protocol, as covariates within a Poisson generalized linear model (GLM). This process regresses out the covariates, and the Pearson residuals are used for factor discovery.
Blinding	No direct sample collection was performed in this study; we relied on the original data study design. The signal extraction is performed in an unsupervised manner, and the resulting factors are then matched with known covariates.

Reporting for specific materials, systems and methods

We require information from authors about some types of materials, experimental systems and methods used in many studies. Here, indicate whether each material, system or method listed is relevant to your study. If you are not sure if a list item applies to your research, read the appropriate section before selecting a response.

Materials & experimental systems

n/a	Involved in the study
☒	☐ Antibodies
☒	☐ Eukaryotic cell lines
☒	☐ Palaeontology and archaeology
☒	☐ Animals and other organisms
☒	☐ Clinical data
☒	☐ Dual use research of concern
☒	☐ Plants

Methods

n/a	Involved in the study
☒	☐ ChIP-seq
☒	☐ Flow cytometry
☒	☐ MRI-based neuroimaging

Plants

Seed stocks	*Report on the source of all seed stocks or other plant material used. If applicable, state the seed stock centre and catalogue number. If plant specimens were collected from the field, describe the collection location, date and sampling procedures.*
Novel plant genotypes	*Describe the methods by which all novel plant genotypes were produced. This includes those generated by transgenic approaches, gene editing, chemical/radiation-based mutagenesis and hybridization. For transgenic lines, describe the transformation method, the number of independent lines analyzed and the generation upon which experiments were performed. For gene-edited lines, describe the editor used, the endogenous sequence targeted for editing, the targeting guide RNA sequence (if applicable) and how the editor was applied.*
Authentication	*Describe any authentication procedures for each seed stock used or novel genotype generated. Describe any experiments used to assess the effect of a mutation and, where applicable, how potential secondary effects (e.g. second site T-DNA insertions, mosiacism, off-target gene editing) were examined.*
